# Carbohydrate-Deficient Transferrin (CDT) as a Biomarker of Alcohol Abuse: A Retrospective Study of the Italian Drinking Trend among Drivers from 2016 to 2022

**DOI:** 10.3390/toxics11110914

**Published:** 2023-11-07

**Authors:** Denise Fiorelli, Leonardo Romani, Michele Treglia, Margherita Pallocci, Pierluigi Passalacqua, Luca Coppeta, Luigi Tonino Marsella, Roberta Tittarelli

**Affiliations:** 1 Laboratory of Forensic Toxicology, Section of Legal and Forensic Medicine, Social Security and Forensic Toxicology, Department of Biomedicine and Prevention, Faculty of Medicine and Surgery, University of Rome “Tor Vergata”, Via Montpellier 1, 00133 Rome, Italy; denise.fiorelli@ptvonline.it (D.F.); roberta.tittarelli@uniroma2.it (R.T.); 2 PhD School in Medical-Surgical Applied Sciences, University of Rome “Tor Vergata”, Via Montpellier 1, 00133 Rome, Italy; 3 Department of Public Health and Infectious Diseases, “Sapienza” University of Rome, Piazzale Aldo Moro 5, 00185 Rome, Italy

**Keywords:** carbohydrate-deficient transferrin (CDT), alcohol abuse, high-performance liquid chromatography (HPLC), driving under the influence of alcohol

## Abstract

Alcohol abuse is still one of the leading causes of death worldwide. Early diagnosis of alcohol abuse enables preventive intervention on the effects and risks associated with its consumption. Carbohydrate-deficient transferrin (CDT) is one of the most reliable biomarkers of chronic alcohol misuse. We retrospectively studied a population of 12,624 subjects who had their driving license suspended for driving under the influence of alcohol or drugs from 2016 to 2022. The analytical determination of CDT was performed following a certified high-performance liquid chromatography (HPLC) method. Data were split by year, age and gender. The majority of subjects with positive %CDT were male, although the trend of positivity was similar between males and females. A steady increase in both the number of tests performed and the number of positives was observed over the years. Patients aged 41–50 years had the highest prevalence, followed by 51–60, 31–40 and 18–30 years. CDT continues to be a steady marker for diagnosis of alcohol abuse in the majority of cases. Data emerging from our study are in line with the increasing national trends on traffic accidents, injuries and deaths related to alcohol and drug DUI (driving under the influence), requiring the implementation of preventive measures to limit this ever-growing phenomenon.

## 1. Introduction

According to the World Health Organization (WHO), alcohol misuse is one of the five factors causing morbidity, disability and mortality worldwide [1,2]. Alcoholism is defined as “a state that, due to alcohol consumption, causes behavioral disturbances, related illnesses or other consequences that could cause present or future harm to the patient, his family or society. Such behavior may or may not be associated with addiction” [3,4].

Excessive and constant alcohol use can cause serious pathologies such as cancer (e.g., oropharynx, esophageal, liver or breast cancer) and gastrointestinal (gastritis, bleeding and ulcers), metabolic (diabetes), liver (e.g., cirrhosis, hepatitis), neuropsychiatric and cardiovascular (myocardial infarction, stroke) diseases.

Heavy alcohol consumption can be ascertained using several biochemical markers. One of the most sensitive and specific serum biomarkers used for determining excessive alcohol consumption is carbohydrate-deficient transferrin (CDT) [5,6].

Serum transferrin (Tf) is a glycoprotein synthesized in the liver that mediates the transport of iron throughout the body. The iron-transport glycoprotein normally has several oligosaccharide chains (N-glycans) with sialic acid end groups, some of which are lost after high alcohol consumption. Based on the number of sialic acid molecules, different transferrin isoforms can be detected: hexa-, penta-, tetra-, tri-, di-, mono- and asialo-Tf. Heavy drinking interferes with glycan synthesis, leading to the loss of sialic acid end groups and to the modification of the Tf glycoform pattern. The excessive consumption of alcohol, protracted over time (more than 50 g of alcohol a day for 1 week), causes an increase in the disialo-transferrin isoform with a consequent increment in the percentage value of CDT (%CDT) [7,8].

The disialo isoform decreases following alcohol abstinence for 14–17 days until reaching a complete normalization in about 3 or 4 weeks [9]. Hence, because of its half-life, CDT is an effective biomarker of long-term heavy alcohol intake on a daily basis, but it cannot be considered as sensitive in cases of occasional drinking or binge drinking (heavy episodic drinking during a short period of time) [10,11].

In recent years, many studies have been published to evaluate the utility of CDT in the diagnosis of alcohol abuse in different countries, compared with other biological markers (gamma-glutamyl transferase (GGT), alanine aminotransferase (ALT), aspartate aminotransferase (AST) and mean cell volume of erythrocytes (MCV)) [12,13,14,15]. The highest sensitivity and specificity of CDT in alcohol-dependent people, 84% and 92%, respectively, were achieved in an Indian population of 25 males with diagnosed heavy alcohol consumption who attended the outpatient department (OPD) of an alcohol de-addiction center of a tertiary care hospital [7,16].

For the identification of alcohol-related pathologies, CDT is mainly used in combination with other alcohol biomarkers, such as GGT and MCV [17,18]. Unlike other biomarkers, CDT is more sensitive to changes in alcohol consumption than to the secondary effects of liver disease, and it is useful to differentiate between alcoholic (ALD) and nonalcoholic fatty liver disease (NAFLD) [19].

The abnormal sialylation pattern can be analyzed using different techniques, including nephelometry, high-performance liquid chromatography (HPLC), capillary electrophoresis (CE) and MALDI-TOF mass spectrometry [20,21].

Several cases of uninterpretable CDT results with abnormal patterns have been described in the literature. Atypical transferrin isoform patterns can be related to the presence of disialo–trisialo bridging (D-TB), a very low transferrin concentration (LT) or an atypical transferrin peak profile (APP) attributable to monoclonal components or other molecules with similar physicochemical properties [22,23,24]. Furthermore, CDT may not be measurable due to the presence of genetic variants (GVs) or carbohydrate-deficient glycoprotein syndromes (CDG I and II), which are rare hereditary glycoprotein disorders.

Moreover, the probability of false negatives or false positives is extremely low in cases of primary biliary cirrhosis, chronic viral hepatitis, hepatocellular carcinoma, hypertransferrinemia, pregnancy, estrogen use, iron deficiency anemia, hypoferritinemia, transplantation and antiepileptic use [25,26,27,28].

The most widespread application of CDT in forensic toxicology concerns the identification of alcohol abuse for determining job suitability and for renewing the driving license of drivers charged with driving under the influence (DUI) of alcohol and/or drugs [29,30,31].

High levels of CDT can be suggestive of recent excessive alcohol intake, so the determination of %CDT is a useful tool for monitoring abstinence in subjects with unhealthy alcohol drinking behaviors or following up a medical detoxification process of those admitted to alcohol rehabilitation units.

In Italy, CDT determination is also required by the Local Medical Committees (LMCs) to screen people whose driving license has been withdrawn for driving under the influence of alcohol or drugs, as a result of road accidents and/or vehicular homicide [32].

Article 186 of the Italian Highway Code in Italy states that drunk driving is forbidden. For drivers with a blood alcohol level equal to or greater than 0.5 g/L (0.05%), the Italian Highway Code establishes the driving ban or cancellation of driving license with administrative and criminal penalties, depending on the blood alcohol concentration (BAC) detected while driving [33].

A special limit of 0.00% applies for new drivers (holding a license for less than 3 years), young drivers aged up to 21 and professional drivers (article 186–bis of the Italian Highway Code) [34].

Victims of road accidents caused by the use of alcohol represent a current and very widespread problem, the incidence of which does not decline despite severe penalties.

The purpose of our retrospective study was to investigate the %CDT data from 2016 to 2022 in a population of 12,624 subjects who had their driving license suspended for driving under the influence of alcohol or drugs.

The data presented in our study clearly highlight the dramatic increase in people having their licenses suspended or revoked for driving while impaired by alcohol or drugs. The results of alcohol-related investigations are an additional source of concern. We first evaluated whether alcohol abuse was associated with gender or a specific age group. Then, we compared our findings with national data with the aim of raising awareness of the risks of drunk-driving accidents and developing effective strategies (e.g., alternative transportation, mandatory driver education, limitation on alcohol sales, the mandatory presence of the alcohol interlock system in cars) to reduce the number of road victims and prevent alcohol-impaired crashes [21].

## 2. Materials and Methods

### 2.1. Subjects and Samples

Blood samples were collected with a Vacutainer system under a strict chain of custody procedure at the Laboratory of Forensic Toxicology, University of Rome “Tor Vergata”, from drivers convicted of driving under the influence of alcohol or drugs coming from Local Medical Committees (LMCs), responsible for physical and mental fitness to drive. A total of 12,624 subjects (88% men and 12% women) attended our laboratory from 2016 to 2022. The tests were mandatory, and the data obtained from CDT tests were aggregated and anonymized as they were collected for non-medical purposes.

In order to diagnose a regular alcohol abuse habit, a wide range of hematological and biochemical parameters are tested. An alteration in liver enzymes (ALT, AST and GGT) and a change in the mean corpuscular volume of red blood cells (MCV) were tested to better evaluate excessive alcohol consumption [35].

For CDT analysis, the blood samples were collected using Vacutainer tubes with a separating gel and coagulation activator. The blood samples were centrifuged at 4500 rpm for 5 min after collection; subsequently, 100 μL of serum was transferred to a 1.5 mL Nerbe Plus microcentrifuge tube, whereas the residual was stored at −20 °C.

### 2.2. HPLC Analysis

In our study, the Eureka srl Lab Division kit (Eureka Lab Division, Sentinel Diagnostics, Chiaravalle, 60033, Italy) was used [36].

A volume of 50 μL of complexing solution (reagent A) was added to the blood collected in the microcentrifuge tubes (100 μL); the sample was then vortexed for 10 s and centrifuged at 10,000 rpm for 10 min. The supernatant was diluted 1:5 (sample: distilled water, total volume 500 μL) in a clear glass vial and injected into the HPLC system.

Separation of transferrin glycoforms was performed on a Thermo Scientific ProPacTM SAX-10G BioLCTM 4 × 50 mm anion-exchange chromatography column (Thermo Fischer Scientific, Waltham, MA, USA) by linear salt gradient elution using an Agilent 1260 Infinity II HPLC system equipped with a G7114A multiple-wavelength detector. Quantification relied on the selective absorbance of the iron–transferrin complex at 460 nm. The relative amount of each glycoform was calculated as a percentage of total transferrin (peak areas for all glycoforms), using the baseline integration mode. To calculate the %CDT, the working group of the International Federation of Clinical Chemistry (IFCC) suggested calculating the percentage of asialo–mono–disialo transferrin in relation to the total transferrin [37,38].

According to the Eureka srl Lab Division kit, samples with a CDT concentration equal to or higher than 2% were considered “positive”, whereas, as reported in the national guidelines, the decision value (cut-off) for CDT concentration is method-dependent [29].

### 2.3. HPLC Methods

The performance of the Eureka Lab Division kit for CDT testing in serum was evaluated on an Agilent 1260 Infinity II HPLC system equipped with a quaternary pump and degasser, a thermostated autosampler (4 °C) and column compartment (35 °C), a multiple-wavelength detector and Chemstation software. The glycoforms were separated on a gradient HPLC system, which involved the use of two mobile phases, M1 and M2, at a constant flow of 1.6 mL/min (Table 1).

### 2.4. Data Analysis

Data analyses and graph preparation were performed with GraphPad Prism 9.1.1 Software (GraphPad Software, San Diego, CA, USA). Categorical data were displayed as numbers and/or percentages, and continuous data were displayed as median and range.

## 3. Results

Our study was performed on blood samples collected from 12,624 drivers convicted of alcohol or drug DUI. The age of the observed population was in the 15–88 age group (mean 51.5, median 37.9).

**Figure 1:**
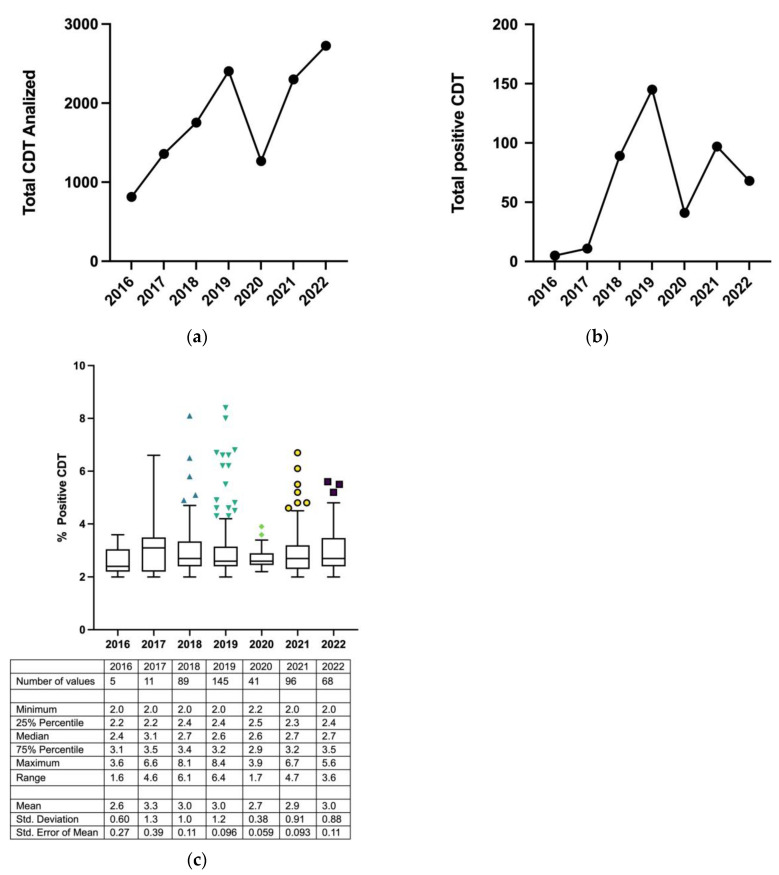
The total number of CDT tests performed (**a**), compared to the positive ones from 2016 to 2022 (**b**). In (**c**), the average percentages of CDT testing positive for each year are given. The symbols in the figure show maximum values.

The data reported in Figure 1a show a progressive increase in the number of CDT tests performed from 2016 (n = 815) to 2019 (n = 2404); a sharp decline was recorded in 2020 (n = 1267), followed by a maximum peak in 2022 (n = 2725). The number of patients with CDT-positive results reached a peak in 2019 (n = 145). Conversely, in the following years (2020–2022), the number of positives was always lower than that in 2019 (2020 n = 41; 2021 n = 97; 2022 n = 68) (Figure 1b).

In Figure 1c, data of the average percentages of CDT testing positive for each year are reported.

**Figure 2:**
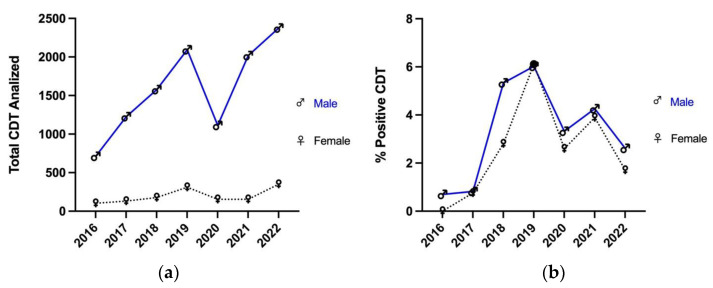
Comparison of the total CDT tests (**a**) with the percentage of positive results, divided by gender from 2016 to 2022 (**b**).

The total CDT tests performed from 2016 to 2022, split by gender, are reported in Figure 2a: the number of males considerably exceeded the number of females, but the trend of the collected data was similar between the groups (Figure 2a).

The male curve increased from 2016 (n = 712) to 2019 (n = 2093), decreased sharply in 2020 (n = 1113) and increased again in 2022 (n = 2375). The trend of the female curve was comparable to the male one: an increase was registered from 2016 (n = 103) to 2019 (n = 311), followed by a decrease in 2020 (n = 154) and new increases in 2021 (n = 283) and 2022 (n = 350).

Figure 2b represents the percentages of males (blue line) and females (dashed black line) with a positive %CDT (≥2%) in relation to the total number of people split by gender from 2016 to 2022.

Our data analysis also focused on the percentage of positive subjects split into different age groups from 2016 to 2022 (Table 2).

**Figure 3:**
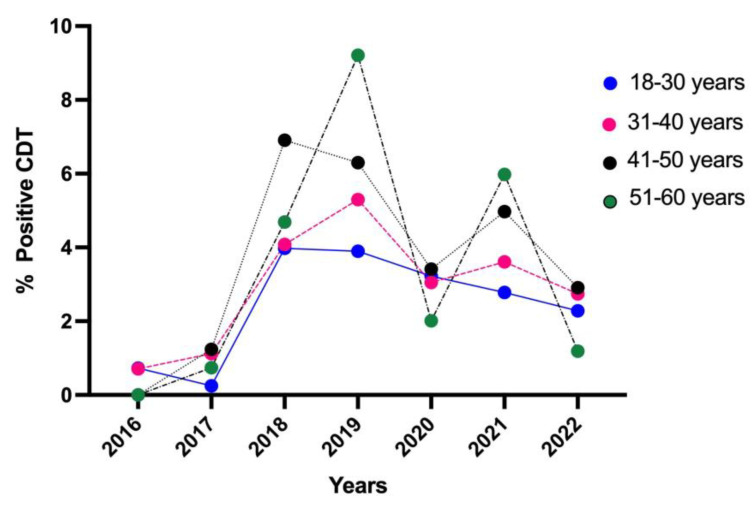
Percentage of CDT-positive results for intermediate age groups from 2016 to 2022. The age groups with a low percentage of CDT-positive results (<18, 61–70 years and >70 years) were excluded because of the low number of subjects.

The curves, shown in Figure 3, had different trends. The age groups with a low percentage of CDT-positive results (<18, 61–70 years and >70 years) were excluded from the graph because of the low number of subjects.

The 18–30 age group (blue line) was characterized by a peak in 2018, followed by a constant decrease until 2022.

In the 31–40 age group (pink line), a progressive increase in the curve was recorded from 2016 to 2019, followed by a decrease in 2020. After a slight increase in 2021, the trend dropped again in 2022.

The age group 41–50 years (black line), had an exponentially growing peak between 2017 and 2018, followed by a rapid decrease starting from 2019. The decrease continued progressively until 2020, followed by an increase in 2021 and a further decrease in 2022.

The last data concern the subjects included in the group aged 51–60 (green line): an oscillating trend can be observed, characterized by an exponential growth peak in 2019, the highest among all the age groups, a sharp decrease in 2020, a marked increase in 2021 and a subsequent decrease in 2022.

### Genetic Tf Variants

In the presence of genetic variants (GV), CDT may not be measurable. The following data (Table 3) allow us to identify CDTs that are not measurable as genetic Tf variants. Our HPLC method does not provide information on the type of variant, so we obtained only useful information about the frequency of genetic variants. A total of 132 genetic variants were observed from 2016 to 2022 (2016 n = 5; 2017 n = 6; 2018 n = 18; 2019 n = 28; 2020 n = 14; 2021 n = 28; 2022 n = 33). For observational purposes, we noted that the majority (n = 123) were males, whereas only nine were females, and the mean male-to-female ratio was 1.25:1 (Table 3). We inferred from the data that there is no link between genetic variants and gender.

Almost all the subjects were Caucasians (83.3%), whereas Balkans (6.1%), Africans (5.3%), Mesoamericans (3.0%) and Chinese (1.5%) represent the remaining part.

**Figure 4:**
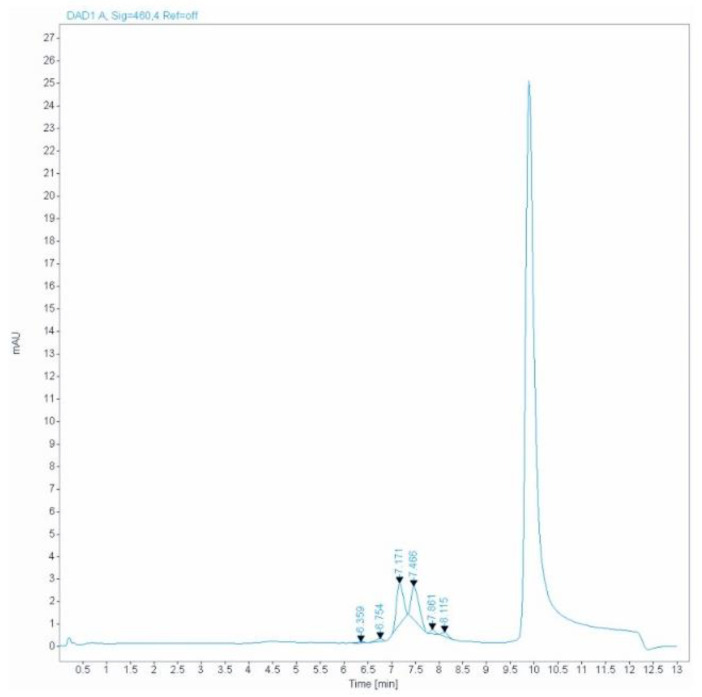
HPLC chromatogram of genetic transferrin variant. Generally, the presence of a genetic variant is easily recognized by a double peak of the major tetrasialotransferrin isoform [37].

We could not exactly identify the type of genetic Tf variants, so we aimed to investigate the genetic characteristics and the frequency of the observed Tf variants in the studied population (Figure 4).

## 4. Discussion

Alcohol consumption is estimated to be responsible for approximately 7.4% of premature deaths and over 200 alcohol-related disabilities in Europe (EU), as well as accidental injuries (e.g., road accidents, poisonings, falls, drowning, burns, fights and interpersonal violence) [39,40,41].

In 2019, the Italian National Statistical Institute (ISTAT) reported that 66.8% of the population in Italy, starting from the age of 11, consumed at least one alcoholic beverage during the year, whereas 20.2% consumed alcoholic beverages every day [42]. A retrospective study of 12,624 CDT tests from 2016 to 2022 was conducted to investigate the trend of alcohol abuse in a population of subjects who had their driving license suspended due to alcohol DUI. The convenience sampling of subjects, although useful for the purpose of our study, is a limitation because the data collected do not represent the whole population.

Data were split by year, age and gender. Furthermore, the prevalence of CDT genetic variants in the studied population was also assessed.

The trend of the curves was similar in the observed years: a decrease in the total number of tests and positive results was observed in 2020, maybe as a consequence of the containment measures for the COVID-19 pandemic, during which there was the closure of venues and a reduction in meeting and socializing opportunities [43]. In 2020, 118,298 road accidents were recorded in Italy, a drastic decrease compared to 2019 (−31.3%) [44]. The number of deaths related to road accidents in Italy was 2395 (−24.5%). The numbers of injured and seriously injured people were also in sharp decline compared to 2019, 159,249 (−34%) and 14,102 (−20%), respectively. Out of 40,310 accidents with injuries, 3692 were caused by drivers in a state of alcohol or drug intoxication and 9.2% and 3.5% of the accidents were attributable to alcohol and drug consumption, respectively.

An exception occurred in 2022, in which our data showed an increase in the total number of subjects and a decrease in the total number of positive CDT tests, probably due to the effectiveness of the prevention policies for the reduction and prevention of driving while intoxicated [45].

In 2022, a sharp upturn in mobility and road accidents occurred, mainly concentrated from January to July. Road accidents, deaths and injuries increased compared to 2021 but were fewer than in 2019 (road accidents and injuries, −3.7% and −7.4%, respectively).

In detail, in 2022 in Italy, 165,889 road accidents occurred (+9.2% compared to 2021), 223,475 people were injured (+9.2%) and 3159 were victims (+9.9%).

The majority of subjects with positive %CDT were males, although the positivity trend was similar over the years between males and females. In particular, our data on positive %CDT for women were in line with the national ones published in 2022 by the Italian National Institute of Health (NIH) [43].

Patients aged 41–50 had the highest prevalence (3.68%), followed by those aged 51–60 (3.40%), 31–40 (2.95%) and 18–30 (2.45%). However, the advent of the COVID-19 pandemic in 2020 led to a decreasing trend in CDT-positive tests, although alcohol e-commerce sales increased worldwide in 2020 [43]. National data on alcohol use during the pandemic showed that 66.4% of Italians consumed at least one alcoholic beverage, with a higher prevalence among men (77.2%) compared to women (56.2%). The highest percentage was recorded in the age group between 18 and 64 for both genders.

Moreover, the study conducted on the frequency of genetic variants showed that a value of 1% of CDT genetic variants was recorded in 2018. The data showed an incidence of Caucasians (83.3%), followed by Balkans (6.1%), Africans (5.3%), Mesoamericans (3.0%) and Chinese (1.5%). The ratio of genetic variants to gender was 1.25% for males compared to 1% for females (1.25:1).

## 5. Conclusions

The harmful consequences of the consumption of alcohol are not limited to the consequences on the subject’s health and psychophysical well-being but extend to the significant social and economic losses related to the costs caused by alcohol (loss of productivity and increase in unemployment).

CDT continues to be a reliable indicator for a diagnosis of alcohol abuse in the majority of cases. Data emerging from our study are in line with the Italian trends. National data on traffic accidents, injuries and deaths related to alcohol and drug DUI are steadily increasing, requiring the implementation of preventive measures to limit this ever-increasing phenomenon.

In this context, the Italian Government, after enacting the law on vehicular homicide in 2016, has recently announced a zero-tolerance reform of the Highway Code with a tightening of penalties for drunk driving. The reform also establishes that any person who drives under the influence of alcohol or drugs commits an offense, with or without psychophysical signs of alcohol or drug impairment.

Moreover, to implement the global strategy to reduce the unhealthy use of alcohol, the World Health Organization (WHO) developed an action plan (2022–2030) with the aim of decreasing alcohol use by 10% in 2025.

Future objectives will be to monitor the response to the implementation of the plan to reduce the use of alcohol following the preventive measures envisaged and to study the type and frequency of Tf genetic variants in the population charged with alcohol-related DUI for driving license regranting.

## Figures and Tables

**Table 1:** Percentage ratio between M1 and M2 at a constant flow of 1.6 mL/min.

Time (min)	%M1 (Pump A)	%M2 (Pump B)
0	100	0
0.5	100	0
5	75	25
5.1	0	100
7.5	0	100
7.6	100	0

**Table 2:** Percentage of positive CDT results per age group from 2016 to 2022.

%Positive CDT							
Age Group	2016	2017	2018	2019	2020	2021	2022
<18	0 (0/0)	0 (0/0)	0 (0/3)	50.0 (1/2)	0 (0/0)	0 (0/2)	0 (0/1)
18–30	0.73 (2/274)	0.25 (1/405)	3.98 (19/477)	3.9 (23/589)	3.22 (10/311)	2.78 (13/467)	2.28 (11/483)
31–40	0.71 (2/283)	1.12 (5/445)	4.08 (22/539)	5.32 (42/789)	3.05 (12/394)	3.61 (28/775)	2.74 (25/911)
41–50	0 (0/182)	1.24 (4/322)	6.91 (32/463)	6.3 (39/619)	3.41 (12/352)	4.97 (30/604)	2.91 (22/757)
51–60	0 (0/59)	0.74 (1/135)	4.69 (10/213)	9.21 (28/304)	2.01 (3/149)	5.98 (21/351)	1.19 (5/421)
61–70	7.14 (1/14)	0 (0/43)	9.8 (5/51)	9.46 (7/74)	6.82 (3/44)	4.6 (4/87)	3.03 (4/132)
>70	0 (0/3)	0 (0/8)	12.5 (1/8)	18.52 (5/27)	5.88 (1/17)	6.67 (1/15)	5 (1/20)

**Table 3:** Number of genetic variants divided by gender from 2016 to 2022.

% of Genetic Variants Split by Gender							
	2016	2017	2018	2019	2020	2021	2022
Males	0.7% (5/712)	0.4% (5/1226)	1% (16/1576)	1.2% (26/2093)	0.9% (10/1113)	1.3% (27/2018)	1.4% (32/2375)
Females	0% (0/103)	0.8% (1/132)	1.1% (2/178)	0.6% (2/311)	2.6% (4/154)	0.4% (1/283)	0.3% (1/350)

## Data Availability

The data presented in this study were obtained from the included studies, and are openly available.
